# The distribution of IgG subclass deposition on renal tissues from patients with anti-glomerular basement membrane disease

**DOI:** 10.1186/1471-2172-14-19

**Published:** 2013-04-15

**Authors:** Zhen Qu, Zhao Cui, Gang Liu, Ming-hui Zhao

**Affiliations:** 1 Renal Division, Department of Medicine, Peking University First Hospital; Institute of Nephrology; Peking University; Key Laboratory of Renal Disease, Ministry of Health of China; Key Laboratory of Chronic Kidney Disease Prevention and Treatment, Ministry of Education, Beijing, China; 2 Peking-Tsinghua Center for Life Sciences, Beijing, China

## Abstract

**Background:**

Renal injury of anti-glomerular basement membrane (GBM) disease is defined by the linear deposition of IgG along GBM and rapidly progressive glomerulonephritis. To date, the distribution of anti-GBM IgG subclasses on renal tissue is still unclear. In the current study, we investigated the deposition of the four IgG subclasses using immunohistochemistry in the renal biopsy specimens from 46 patients with anti-GBM disease.

**Results:**

All four IgG subclasses can be detected within the GBM. Anti-GBM IgG3 was detected in all patients (100%), with 39 (84.8%) patients presenting with weak segmental staining and 7 (15.2%) patients with strong linear deposition. Anti-GBM IgG2 was detected in 22 (47.8%) patients, with 20 (90.9%) patients having weak segmental deposition and 2 (9.1%) patients presenting strong linear staining. Anti-GBM IgG1 and IgG4 were detected in 9 (19.6%) and 7 (15.2%) patients, respectively. IgG deposition along tubular basement membrane (TBM) was also detected in 31 (67.4%) patients. Among them, the IgG subclass distribution was similar to that of the deposition within the GBM: IgG1 6.5% (2/31), IgG2 45.2% (14/31), IgG3 100% (31/31) and IgG4 9.7% (3/31). We observed increased inflammatory cell infiltration into the interstitium in patients with increased anti-TBM IgG3 deposits (*P*=0.031).

**Conclusions:**

Anti-GBM IgG3 predominantly deposits along GBM and TBM on renal biopsy specimens from patients with anti-GBM disease, which may be involved in the development of renal injury of the disease.

## Background

Anti-glomerular basement membrane (GBM) disease, also known as Goodpasture’s disease, is an organ specific autoimmune disorder characterized by rapidly progressive glomerulonephritis, pulmonary hemorrhage and presentation of anti-GBM autoantibodies [1]. Linear deposition of immunoglobulin G (IgG) along GBM is the hallmark of anti-GBM glomerulonephritis, and is occasionally accompanied by deposition along tubular basement membrane (TBM). The pathogenicity of anti-GBM IgG has been demonstrated in passive transfer studies using the renal elute taken from patients, which is subsequently injected into squirrel monkeys. The authors observed a similar linear deposition of IgG along the GBM, which contributed to the development of crescentic glomerulonephritis [2]. The target antigen has been identified as the non-collagenous domain (NC1) of α3 chain of type IV collagen on GBM [α3(IV)NC1]. There are two major conformational epitopes within the antigen, E_A_ and E_B_, which are sequestrated in the quaternary structure of GBM by critical sulfilimine bond [3,4].

Human IgG are categorized into four subclasses (IgG1, IgG2, IgG3 and IgG4) according to their heavy chains, which elicit different immunological and inflammatory responses. Previous studies have reported that there is anti-GBM IgG subclass restriction in the circulation of patients with anti-GBM disease, in which IgG1 and IgG4 were predominant [5]. Our recent studies showed that during the initiation and progression of anti-GBM glomerulonephritis, there is an attendant increase of frequencies of anti-GBM IgG1 and IgG3 [6]. This correlation highlights the pathogenic role of IgG1 and IgG3 in the development of renal injury. Animal models also showed that the severity of anti-GBM antibodies induced glomerulonephritis was dependent on the different types of IgG subclasses [7]. However, in human anti-GBM disease, IgG subclass distribution along GBM has not been clearly elucidated, and their association with disease severity remains elusive.

The aim of the current study is to investigate the distribution of IgG subclass deposition along GBM and TBM on renal biopsy specimens from patients with anti-GBM disease in different clinical spectrum. Their association with the clinical and pathological parameters and the outcomes of patients was further explored.

## Results

### Demographic and clinical data of patients

The demographic, clinical and pathological parameters of the 46 patients with anti-GBM disease are listed in Table 1.

**Table 1:** Demographic, clinical parameters and outcomes of patients with anti-GBM disease

	**n=46**
Age (years)	35.3±15.7
Gender (male/female)	32/14
Hydrocarbon exposure, n (%)	3/44 (6.8%)
Prodromal infection, n (%)	19/46 (41.3%)
Smoking, n (%)	24/45 (53.3%)
Hemoptysis, n (%)	15/46 (32.6%)
Oliguria/anuria, n (%)	14/46 (30.4%)
Gross hematuria, n (%)	15/31 (32.6%)
Nephrotic syndrome, n (%)	19/31 (61.3%)
Serum creatinine at presentation (μmol/L)	728.5±378.8
Level of anti-GBM antibodies (U/mL) median (range)	63 (15, 176)
Positive with ANCA, n (%)	9/46 (19.6%)
Hemoglobin (g/L)	85.6±26.1
Percentage of crescents in glomeruli (%)	78.8±29.6
Cellular crescents (%)	60.3±33.8
Fibrotic crescents (%)	33.2±31.0
Renal survival (1 year), n (%)	10/46 (21.7%)
Patient survival (1 year), n (%)	36/46 (78.3%)

GBM: glomerular basement membrane; ANCA: anti-neutrophil cytoplasmic antibody.

Of the 46 patients, the average age was 35.3±15.7 years, with a male to female ratio of 2.3:1 (32 male and 14 female). Fifteen (32.6%) patients had pulmonary hemorrhage and presented as Goodpasture’s syndrome. The mean level of serum creatinine at presentation was 728.5±378.8 μmol/L. The median level of serum anti-GBM antibodies was 63U/mL (range from 15 to 176U/mL, normal <13U/mL). Nine (19.6%) patients were ANCA positive, with 8 specific to MPO and 1 specific to PR3.

On renal biopsy, an average of 22±9.8 glomeruli could be seen in each specimen. 78.8±29.6% of the glomeruli formed crescent and 41 (89.1%) patients were diagnosed of crescentic glomerulonephritis. The percentage of crescents in glomeruli was positively correlated with the level of serum creatinine on diagnosis (r=0.637, *P*<0.001). Linear IgG immunofluorescence along GBM was demonstrated for each of the 46 patients with staining intensity of 1+~4+ on the scale of 1 to 4, in the absence of fluorescence for albumin or a diagnosis of diabetes. Complement (C) 3 was detected in 26 patients (26/46, 56.5%). The staining intensity of IgG along GBM was correlated with that of C3 (r=0.456, *P*=0.002). The staining intensity of neither IgG nor C3 presented correlation with the percentage of cellular cresents (for IgG: *P*=0.322; for C3: *P*=0.635) or fibrotic crescents (for IgG: *P*=0.596; for C3: *P*=0.272).

### The distribution of IgG subclass deposition along GBM

**Figure 1:**
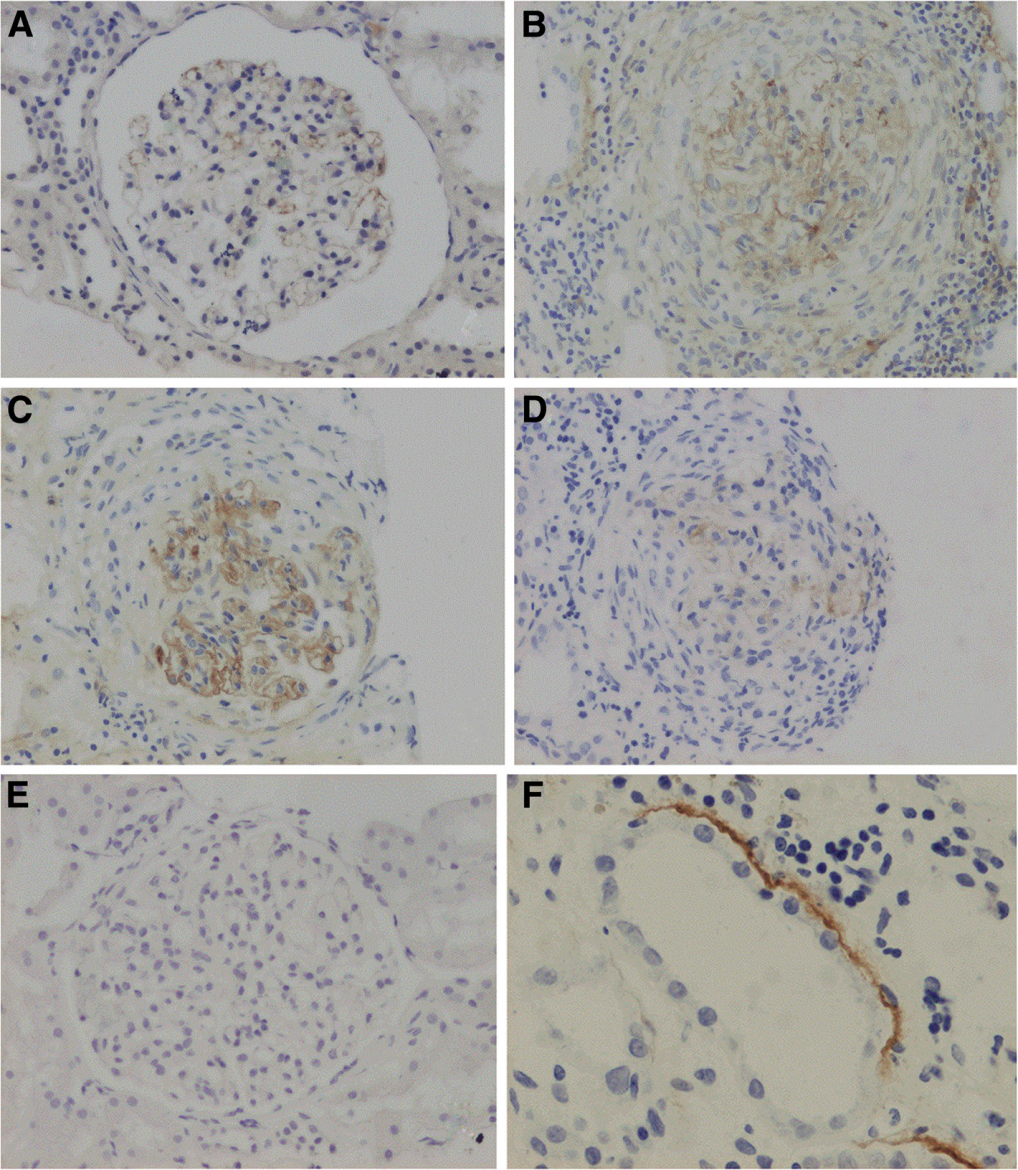
**Immunohistochemistry for IgG subclasses on renal biopsy sections from patients with anti-GBM disease (×400). A.** The weakly segmental deposition of IgG1 along GBM; **B.** The strong linear deposition of IgG2 along GBM; **C.** The strong linear deposition of IgG3 along GBM; **D.** The weakly linear deposition of IgG4 along GBM; **E.** Negative control. F. IgG3 partially deposits along TBM and inflammatory cell infiltration in the interstitium near TBM.

All the four IgG subclasses deposition along GBM were detected on renal biopsy sections from the 46 patients (Table 2) (Figure 1).

**Table 2:** Distribution and intensity of each IgG subclass deposition along GBM and TBM from patients with anti-GBM disease

	**Negative**	**Weak/Segmental**	**Strong/Linear**	**All positive**
Glomerular basement membrane				
IgG1	37 (80.4%)	9 (19.6%)	0	9 (19.6%)
IgG2	24 (52.2%)	20 (43.5%)	2 (4.3%)	22 (47.8%)
IgG3	0	39 (84.8%)	7 (15.2%)	46 (100%)
IgG4	39 (84.8%)	6 (13%)	1 (2.2%)	7 (15.2%)
Tubular basement membrane				
IgG1	44 (95.7%)	1 (2.2%)	1 (2.2%)	2 (4.4%)
IgG2	32 (69.6%)	10 (21.7%)	4 (8.7%)	14 (30.4%)
IgG3	15 (32.6%)	23 (50%)	8 (17.4%)	31 (67.4%)
IgG4	43 (93.5%)	2 (4.3%)	1 (2.2%)	3 (6.5%)

Anti-GBM IgG3 was observed along GBM on renal sections in all the 46 patients (100%). Thirty-nine (84.8%) patients showed weak and segmental staining on glomeruli, and 7 (15.2%) patients showed strong and linear deposition.

Anti-GBM IgG2 was found on glomeruli in 22 (47.8%) patients, with 20 (90.9%) patients having weak and segmental deposition and 2 (9.1%) patients presenting strong and linear staining.

Anti-GBM IgG1 and IgG4 were less abundant on the glomeruli. For IgG1, only 9 (19.6%) renal samples stained positive and the intensity was weak, spotty and segmental staining along GBM. For IgG4, only 7 (15.2%) patients were detected of positive staining, with 1 displaying a strong linear deposition along the GBM.

No correlation was found among the four IgG subclasses of their distribution and intensity along GBM (*P*>0.05).

### The correlation between IgG subclass deposition along GBM and clinical parameters

Correlation between the deposition of each IgG subclass along GBM and the clinical, pathological data and outcomes of patients were analyzed, including age, gender, exposure to hydrocarbon, smoking, serum level of anti-GBM antibodies, positive ANCA, prevalence of nephrotic syndrome, hematuria and oliguria/anuria, serum creatinine at presentation, renal histopathology, and the renal/patient outcomes (Table 3).

**Table 3:** Correlation between the deposition of IgG subclasses along GBM and the clinical and pathological parameters of patients

	**IgG1**			**IgG2**			**IgG3**			**IgG4**		
	**Positive (n=9)**	**Negative (n=37)**	***P***	**Positive (n=22)**	**Negative (n=24)**	***P***	**Strong/Linear (n=7)**	**Weak/Segmental (n=39)**	***P***	**Positive (n=7)**	**Negative (n=39)**	***P***
Age (years)	38.8±13.8	34.5±16.2	0.286	38.1±15.6	32.7±15.7	0.202	37.6±10.4	34.9±16.6	0.320	31.4±16.1	36±15.7	0.529
Gender (male/female)	5/4	27/10	0.308	15/7	17/7	0.845	5/2	27/12	0.907	6/1	26/13	0.313
Hydrocarbon exposure, n (%)	1/9 (11.1%)	2/35 (5.7%)	0.567	1/21 (4.8%)	2/23 (8.7%)	0.605	1/7 (14.3%)	2/37 (5.4%)	0.393	0/7 (0%)	3/37 (8.1%)	0.435
Prodromal infection, n (%)	6/9(66.7%)	13/37 (35.1%)	0.085	8/22 (36.4%)	11/24 (45.8%)	0.515	3/7 (42.9%)	16/39 (41.0%)	0.928	2/7 (28.6%)	17/39 (43.6%)	0.457
Smoking, n (%)	4/8 (50%)	20/37 (54.1%)	0.835	12/21 (57.1%)	12/24 (50%)	0.632	4/7 (57.1%)	20/38 (52.6%)	0.826	4/7 (57.1%)	20/8 (52.6%)	0.826
Gross hematuria, n (%)	2/9 (22.2%)	13/37 (35.1%)	0.459	9/22 (40.9%)	6/24 25%)	0.250	0/7 (0%)	15/39 (38.5%)	**0.046**	3/7 (42.9%)	12/39 (30.8%)	0.530
Nephrotic syndrome, n (%)	3/5(60.0%)	16/26 (61.5%)	0.948	8/14 (57.1%)	11/17 (64.7%)	0.667	4/6 (66.7%)	15/25 (60%)	0.763	4/5 (80%)	15/26(57.7%)	0.348
Hemoglobin (g/L)	94±28.5	83.5±25.2	0.493	90.6±24.2	80.9±27.6	0.146	105.1±25.8	82.0±24.9	**0.038**	84.3±29.7	85.9±25.9	0.818
Oliguria/anuria, n (%)	3/9(33.3%)	11/37 (29.7%)	0.833	7/22 (31.8%)	7/24 (29.2%)	0.845	0/7 (0%)	14/39 (35.9%)	0.057	2/7 (28.6%)	12/39 (30.8%)	0.907
Serum creatinine at presentation (μmol/L)	503.8±341.9	783.2±371.0	0.031	674.9±373.7	777.8±384.6	0.350	445.4±333.0	779.4±367.4	**0.028**	825±532.6	711.2±350.8	0.490
Anti-GBM antibodies (U/mL)	56.1±34.9	69.7±40.8	0.359	90.6±24.2	80.9±27.6	0.452	39.8±42.9	70±38.7	0.060	64.5±59.5	67.4±36.3	0.566
ANCA, n (%)	2/9 (22.2%)	7/37 (18.9%)	0.823	4/18 (22.2%)	5/24 (20.8%)	0.821	0/9 (0%)	9/39 (23.1%)	0.156	2/7 (28.6%)	7/39 (17.9%)	0.514
Percentage of total crescents (%)	60.6±44.3	832±23.6	0.397	76.4±30.1	81.0±29.7	0.587	54.7±47.0	83.1±23.8	0.183	68.9±38.7	80.5±28	0.719
Cellular crescents (%)	43.3±36.1	64.5±32.3	0.086	65.3±34.0	55.8±33.6	0.285	39.6±34.8	64.0±32.6	0.093	58.9±38.6	60.6±33.4	1
Fibrotic crescents (%)	34.5±32.9	32.8±30.9	0.892	25.6±27.9	40.1±32.5	0.113	31.8±30.8	33.4±31.4	0.858	41.1±38.6	31.7±29.8	0.590
C3 deposits (median)	2	1	1.000	0	1	0.627	2	1	0.131	2	1	0.131
Ultrastructrual lesions	2	2	0.438	2	2	0.727	2	2	0.438	2	2	0.438
Renal survival (1 year), n (%)	3/9 (33.3%)	7/37 (18.9%)	0.347	5/22 (22.7%)	5/24 (20.8%)	0.876	4/7 (57.1%)	7/39 (17.9%)	0.141	1/7 (14.3%)	9/39 (23.1%)	0.604
Patient survival (1 year), n (%)	6/9 (66.7%)	30/37 (81.1%)	0.347	17/22 (77.3%)	19/24 (79.2%)	0.876	7/7 (100%)	29/39 (74.4%)	0.130	4/7 (57.1%)	32/39 (82.1%)	0.141

GBM: glomerular basement membrane; ANCA: anti-neutrophil cytoplasmic antibody.

We found that the patients with strong linear staining of IgG3 along GBM presented with lower prevalence of gross hematuria (0% vs. 38.5%, *P*=0.046), higher level of hemoglobin (105.1±25.8 vs. 82.0±24.9 g/L, *P*=0.038), and lower level of serum creatinine (445.4±333.0 vs. 779.4±367.4 μmol/L, *P*=0.028), compared with those with weak staining of IgG3. Although not statistically significance, the levels of anti-GBM antibodies also seemed to be lower in patients with strong IgG3 deposition than those with weak deposit (39.8±42.9 vs 70.0±38.7U/mL, *P*=0.060).

No correlation was found between the GBM deposition of IgG1, IgG2 or IgG4 subclass and the clinical, pathological parameters or outcomes of patients with anti-GBM disease (*P*>0.05).

### The distribution of IgG subclass deposition along TBM

Anti-GBM IgG deposition along TBM could be detected in 31 (67.4%) patients (Table 2, Figure 1). Among these patients, the IgG subclass distribution along TBM was similar to that of the GBM: IgG1 6.5% (2/31), IgG2 45.2% (14/31), IgG3 100% (31/31) and IgG4 9.7% (3/31). No correlation was found between the IgG subclass distribution along TBM and the clinical data or the outcomes of patients (*P*>0.05). The degree of interstitial inflammation was more severe in patients with stronger deposits of IgG3 along TBM (*P*=0.031, Table 4).

**Table 4:** The severity of inflammatory cell infiltration in interstitium in patients with different intensity of IgG3 deposits along TBM

**Severity of inflammatory cell infiltration**	**Score 1 (n=7)**	**Score 2 (n=10)**	**Score 3 (n=17)**	**Score 4 (n=12)**
Patients without TBM-IgG3	4	3	2	6
Patients with mild deposited TBM-IgG3	3	7	9	4
Patients with strong deposited TBM-IgG3	0	0	6	2

### The correlation between IgG subclass deposition on GBM and their level in the sera

Out of 46 patients whose renal specimens were examined, serum from 32 individuals were collected and tested for IgG subclass distribution in circulation. The positive rate of IgG1, IgG2, IgG3 and IgG4 were 87.5% (28/32), 81.25% (26/32), 75% (24/32) and 81.25% (26/32), with the average level of 0.77±0.90, 0.23±0.36, 0.17±0.23 and 0.61±0.94, respectively. We analyzed the correlation between the serum antibody levels and the intensity of IgG subclass deposition on GBM and TBM. No relations was found between them (*P*>0.05).

## Discussion

In the current study, we examined the distribution of IgG subclass deposition along GBM and TBM on renal biopsy specimens from a large cohort of 46 patients with anti-GBM disease. We found that all four IgG subclasses could be detected with vastly different distributions. For the deposition on the GBM, IgG3 was detected in all patients with mild segmental to strong linear staining. IgG2 was found in approximately half of the patients. IgG1 was only detected in one fourth of the patients and the intensity of deposition was weak and segmental. IgG4 was the least abundant subclass detected on the GBM.

The general detection of IgG3 along the GBM and the TBM suggests that it is likely to play an important role in the pathogenesis of renal lesions, which contribute to the development of human anti-GBM disease. The structural characteristics that distinguish the four subclasses are the size of the hinge region and the number and position of the interchain disulfide bonds between heavy chains. With an elongated 62-amino-acid hinge region, IgG3 molecules are the most effective complement activator [8]. Therefore, Clq could bind more readily to the Fc region of IgG3 and thus facilitate the complement-associated inflammatory response. The general finding of linear or granular C3 deposition accompanying IgG on GBM supports the activation of complement system in glomeruli during the progress of anti-GBM nephritis [9,10]. Another mechanism for IgG3 inducing tissue damage lies in its high affinity binding to Fc receptors (R) on phagocytic cells to mediate opsonization [11]. The IgG Fc-FcR interaction in glomeruli could also mediate the accumulation of macrophages to induce renal injury [12]. Accelerated models of anti-GBM glomerulonephritis induced in FcγRI−/−, FcγRIII−/− and FcRγ−/− mice have demonstrated that FcRγ, especially FcγRIII, plays a pivotal role in the pathogenesis through the antibody-dependent pathway [13]. Moreover, the copy number variation of *FCGR3A*[14] and the gene polymorphism of *FCGR2B*[15] have been demonstrated as contributors to the susceptibility of anti-GBM disease in Chinese population. These mechanisms may be involved in the pathogenesis of renal injury after IgG3 deposition along GBM and TBM.

In our study, we also detected the circulating IgG subclasses against rHα3(IV)NC1, which showed that all the four subclasses were presented. In comparison with our previous study [6], we found that most patients from our present study had severe renal damage, with all the four IgG subclasses in circulation. The level of circulating IgG subclasses was not correlated with the deposition of IgG subclasses on renal tissue. The predominant of IgG3 deposition on glomeruli is in contrast with previous reports about the anti-GBM IgG subclass distribution in circulation [5,16]. It is demonstrated that anti-GBM IgG in sera is mainly of IgG1 (94%) and IgG4 (88%) subclasses. IgG3 was found in only 12% of the sera from patients [16]. We assume it might be difficult to detect IgG3 in the serum because of its short life time (8 days), but it located on glomeruli and induced damage with formation of crescents. Although it had no statistical significance, the circulating IgG1 and tissue IgG1 showed a mild correlation, which *P*=0.107, which might be contributed to the longer life time of IgG1 in circulation (23 days) for easier detection [8]. Despite its deficiency in sera, the close relationship between IgG3 and the progression of human anti-GBM nephritis was proven, as IgG3 being absent in natural anti-GBM antibodies from healthy individuals [7,17], while occurring in patients with renal dysfunction [6]. Animal experiments also showed that monoclonal IgG2a and IgG2b (equivalent to human IgG1 and IgG3 [18]) against α3(IV)NC1 could induce severe nephritis and pulmonary hemorrhage in rats [19]. As an organ-specific autoimmune disease, the immune response of anti-GBM autoantibodies is directed to the target antigen in situ of the glomerular and alveolar basement membrane. Thus, the target organs may be damaged much directly by humoral or cellular mechanisms in the local immunity rather than the systemic immunity.

The correlation between the IgG subclass deposition and the clinical and pathological parameters of patients was analyzed in the current study. It was shown that patients with strong linear deposition of IgG3 on GBM presented with milder disease severity with lower serum creatinine and lower circulating anti-GBM antibodies on diagnosis. This finding emphasizes the important role of IgG3 in the early stage of kidney impairment in human anti-GBM disease. Cellular immunity has also been demonstrated to participate in the formation of severe crescentic glomerulonephritis [20]. It may be speculated that the structure of glomerular capillary walls are severely damaged and fractured in the later stage of crescent formation, which may result in the deposition of IgG showing weaker segmental staining in more severe renal lesions. This may also be the explanation for the different findings from Noël et al., who found that IgG subclass deposited in glomeruli were mostly IgG1 and IgG4 [21]. The discrepancies might be explained by the different demographics and ethnicity of patients, different detecting monoclonal antibodies and differences in investigating approaches. In our study, we performed immunohistochemistry instead of immunofluorescence, which was employed by the previous study (Noël et al. [21]). We both utilized direct immunofluorescence with strict positive and negative controls. However, the large number of patients in the current study contains patients with different stages of renal impairment, i.e. from the mildest, to severe kidney dysfunction requiring dialysis, provided better opportunities to reveal the distribution of IgG subclasses in a wide spectrum of kidney lesions.

IgG deposition on TBM was also detected in 67.4% of the patients, with accordant distribution as it was along GBM. TBM deposition is not common in anti-GBM nephritis [22]. We suggested that the high detection rate of IgG deposits along TBM may be due to the high sensitivity of immunohistochemistry used in the current study. Using this method, we further found that the inflammatory cell infiltration in interstitium was more severe in patients with strong anti-TBM IgG3 deposits, which implies that the deposition of autoantibodies on TBM may contribute to the inflammatory cell infiltration in renal interstitial and/or causes direct tubular damage.

## Conclusions

Anti-GBM IgG3 predominantly deposits along GBM and TBM on renal biopsy specimens from patients with anti-GBM disease, which may play a pivotal role in the development of anti-GBM glomerulonephritis.

## Methods

### Patients

Forty-six patients with renal biopsy-proven anti-GBM disease diagnosed in Peking University First Hospital from 1994 to 2008 were enrolled in this study. They were diagnosed in our hospital or referred to our hospital by physicians or nephrologists from other hospitals throughout China. The clinical manifestations and pathologic data were collected from medical records at the time of presentation and during follow-up. The research was in compliance with the Declaration of Helsinki and approved by the ethics committee of our hospital (reference number 418).

### Detection of anti-GBM antibodies and ANCA

Sera from all patients were screened at presentation before the initiation of immunosuppressive treatment. Anti-GBM assays were performed by enzyme-linked immunosorbent assay (ELISA) using purified bovine α(IV)NC1 as solid phase antigen, with confirmation of antibody specificity by ELISA against recombinant human α3(IV)NC1. Anti-neutrophil cytoplasmic antibody (ANCA) assays were performed by indirect immunofluorescence (EUROIMMUN, Lübeck, Germany) using ethanol-fixed human neutrophils. Antigen-specific ELISA was performed against purified myeloperoxidase (MPO) and proteinase 3 (PR3).

### Renal histopathology

Renal biopsy was performed at the time of diagnosis. Renal specimens were evaluated using direct immunofluorescence, light and electron microscopy and were forwarded to two pathologists. The biopsies were independently assessed in a blinded manner by two pathologists.

For direct immunofluorescence, frozen sections were examined by a fluorescent microscope (Nikon, Tokyo, Japan) after staining with fluorescein isothiocyanate (FITC)-conjugated antibodies specific for human IgG, IgM, IgA, C3, C1q, fibrinogen and albumin (Dako, Copenhagen, Denmark). The fluorescence was semi-quantitatively graded from 0 to 4+ according to the intensity. For light microscopy, consecutive series of 4 μm paraffin sections were stained with haematoxylin and eosin, periodic acid-Schiff with silver methenamine and Masson’s trichrome. For electron microscopy, biopsy specimens were fixed in 2.5% glutaraldehyde, post-fixed in 1% osmium tetroxide. Then it was dehydrated in graded acetone, and embedded in Epon 812 resin. Ultrathin sections were stained with uranyl acetate and lead citrate, examined by a transmission electron microscope (JEM-1230, JEOL, Tokyo, Japan).

The glomerular injury was evaluated by the percentage of crescents in glomeruli. The tubular-interstitial injury was scored 1~4 based on the involved area, as score 1 (<25%), score 2 (25~50%), score 3 (50~75%) and score 4 (>75%). The ultrastructural lesion were scored based on the crescent formation and inflammatory cell infiltration in glomeruli, as score 1 for mild injury and score 2 for severe injury.

### Immunohistochemistry for anti-GBM IgG subclasses on renal biopsy sections

Paraffin-embedded specimens of formalin-fixed renal biopsy tissue were processed for immunohistochemical staining for IgG subclasses using mouse monoclonal antibodies against human IgG1, IgG2, IgG3 and IgG4 (γ chains specific, Southern Biotech, Birmingham, USA). The clone numbers were 4E3, HP6014, HP6050 and HP6025, respectively. The optimal time and temperature for incubation were predetermined. Renal sections were treated at room temperature firstly with 0.4% pepsin (Zhongshan Golden Bride Biotechnology, Beijing, China), 30 min, for antigen retrieval, then with freshly prepared 3% hydrogen peroxide in methanol solution, 20 min, to quench endogenous peroxidase activity, and last 1% bovine serum albumin (BSA) at 37°C for 30 min to block non-specific staining. Monoclonal antibodies were diluted 1:100 in 0.01 mol/L phosphate buffered saline (PBS), pH 7.4, for incubation at 4°C over night. The secondary antibodies of goat anti-mouse immunoglobulins conjugated with horseradish peroxidase (Dako, Copenhagen, Denmark) were diluted 1:100 and incubated at 37°C for 1 h. Then, the sections were developed in fresh hydrogen peroxide plus 3-3-diaminobenzidine tetrahydrochloride solution for 30 sec. Renal sections from patients with membranous nephropathy and membranous lupus nephritis were used as positive controls, normal renal sections and those from patients with minimal change disease and thin basement membrane disease were used as negative controls, PBS was used as blank in replacement for monoclonal antibodies. The extensity and intensity of staining was evaluated at ×400 magnification and scored semiquantitatively: 0, no staining; 1, weakly, spotty and segmental staining; 2, moderate or strong linear staining.

### Detection of anti-GBM IgG subclasses in sera by ELISA

Detection of anti-GBM IgG subclasses by ELISA is performed as previously described [6]. The rHα3(IV)NC1 was coated at 2 μg/ml, the test sera were diluted at 1:50 with PBS containing 0.1% Tween 20, and the horseradish peroxidase-conjugated monoclonal mouse anti-human IgG1, IgG2, IgG3 and IgG4 (Clone No.4E3, HP6014, HP6050 and HP6025) antibodies were diluted at 1:500. The results were recorded as the net OD 405 nm (Bio-Rad, Tokyo, Japan) (the average value of antigen coated wells minus the average value of antigen-free wells).

### Statistical analysis

Differences of quantitative parameters were assessed using the Student *t* test (for data that were normally distributed) or nonparametric test (for data that were not normally distributed). Differences of semi-quantitative data were tested using Mann–Whitney U test. Differences of qualitative data were compared using chi-square test or Fisher’s exact tests. Spearman’s correlation test was used to measure the correlation between two non-parametric variables or one non-parametric variable with one parametric variable. Statistical significance was considered as *P*<0.05. Analysis was performed using SPSS statistical software package (version 13.0, SPSS, Chicago, IL, USA).

## Competing interests

All authors declare that they have no competing interests.

## Authors’ contributions

ZQ carried out the immunohistochemistry staining of the subclass of IgG, and drafted the manuscript. ZC, contributed to collection of data, and revised the critical content of manuscript. GL, contributed to reevaluated the immunohistochemistry staining of the subclass of IgG as a renal pathologist. MZ conceived of the study and participated in its design and coordination and helped to draft the manuscript. All authors read and approved the final manuscript.
